# Non-HLA Autoantibody-Driven Lung Allograft Injury, Clinical Course, Autoantibody Profiling, and Response to Steroid and Belatacept

**DOI:** 10.3390/ijms27219424

**Published:** 2026-10-22

**Authors:** Annika Sundlof, Michiko Taniguchi, Rohit Gupta, Mehri Mollaee, Francis Cordova, Kartik Shenoy, Steven Geier, Abdelhamid Liacini

**Affiliations:** 1 Department of Internal Medicine, Temple University Hospital, Philadelphia, PA 19140, USA; 2 Department of Pathology and Laboratory Medicine, Lewis Katz School of Medicine, Philadelphia, PA 19140, USA; 3 Department of Pathology and Immunology, Washington University School of Medicine, Saint Louis, MO 63110, USA; 4 Immunogenetics Laboratory, Temple University Hospital, Philadelphia, PA 19140, USA; 5 Department of Thoracic Medicine and Surgery, Temple University Hospital, Lewis Katz School of Medicine, Philadelphia, PA 19140, USA; 6 Histocompatibility Laboratory Services, American National Red Cross, 180 Rustcraft Road, Dedham, MA 02026, USA

**Keywords:** lung transplantation, autoantibodies, rejection, antibody-mediated rejection (AMR), AT1R

## Abstract

The presence of HLA donor-specific antibodies (DSAs), both before and after transplantation, is a well-established risk factor for antibody-mediated rejection (AMR) and poor graft survival. However, emerging evidence suggests that non-HLA antibodies may also provoke immune responses, contributing to rejection. Among these, antibodies targeting the angiotensin II type-1 receptor (AT1R) have been linked to adverse clinical outcomes in kidney and orthotopic heart transplant recipients. This case presents an investigation of non-HLA antibodies in a lung transplant recipient diagnosed with AMR in the absence of HLA DSAs, as well as the use of belatacept as part of a combination immunosuppressive regimen to treat AMR in a lung transplant recipient.

## 1. Introduction

Lung transplantation remains a life-saving treatment for patients with end-stage pulmonary disease; however, long-term graft survival continues to be limited by immune-mediated injury and chronic allograft dysfunction [1]. Human leukocyte antigen (HLA) donor-specific antibodies (DSAs), present either before or after transplantation, are well-established risk factors for antibody-mediated rejection (AMR), chronic lung allograft dysfunction (CLAD), and reduced graft survival [2,3]. Nevertheless, HLA-DSAs do not account for all episodes of graft dysfunction, suggesting that additional immunologic mechanisms contribute to allograft injury [1,2,3].

Increasing evidence supports a pathogenic role for non-HLA antibodies directed against endothelial, epithelial, and extracellular matrix antigens, including angiotensin II type-1 receptor (AT1R), MHC class I polypeptide-related sequence A (MICA), and anti-endothelial cell antibodies (AECAs) [4,5,6,7,8,9,10,11,12,13,14,15,16,17,18,19] (Table 1). Unlike conventional HLA antibodies, anti-AT1R antibodies are agonistic in nature, binding and activating the angiotensin II type-1 receptor. This interaction initiates intracellular signaling pathways involving Rho GTPases, ERK1/2, NF-κB, and MAP kinases, leading to endothelial dysfunction, cytokine release, leukocyte recruitment, vasoconstriction, and tissue fibrosis [4,11,20]. As a result, anti-AT1R antibodies may induce graft injury through predominantly complement-independent mechanisms, distinguishing them from many HLA-mediated responses [4,11,20].

The interplay between alloimmunity and autoimmunity is increasingly recognized as an important contributor to transplant rejection. Ischemia–reperfusion injury, infection, and persistent inflammation may expose normally sequestered self-antigens, resulting in loss of immune tolerance and the development of autoantibodies against targets such as vimentin, collagen V, K-alpha-1 tubulin, and LG3 [7,8,9,10,12,21,22,23]. These immune responses can establish self-perpetuating inflammatory and profibrotic pathways that contribute to allograft injury even in the absence of detectable HLA antibodies [11,12].

Several studies have identified pre-existing and de novo anti-AT1R antibodies as risk factors for primary graft dysfunction, chronic rejection, and inferior clinical outcomes following lung transplantation [4,6,7,8,9,10]. However, the optimal management of non-HLA antibody-mediated rejection remains poorly defined. Belatacept, a selective co-stimulation blocker that inhibits T-cell activation through CD80/CD86-CD28 pathway blockade, has shown promise in the treatment of AMR in kidney transplantation, but its role in lung transplant recipients remains under investigation [24,25,26,27].

Here, we describe a case of AMR associated with non-HLA antibodies in a lung transplant recipient in the absence of detectable HLA-DSAs. This case highlights the potential clinical value of non-HLA antibody screening in selected patients with unexplained graft dysfunction and illustrates the use of belatacept as part of a multimodal therapeutic strategy for non-HLA-mediated AMR. Non-HLA antibody results are presented as mean fluorescence intensity (MFI) values obtained using the manufacturer’s assay platform. MFI values reflect relative antibody-binding strength and should not be interpreted as direct quantitative antibody concentrations.

## 2. Case Presentation

A 61-year-old unsensitized male patient (calculated panel reactive antibody [cPRA] 0%) underwent single lung transplantation (LTx) for end-stage interstitial lung disease. No HLA antibodies were detected by FlowPRA or single antigen beads (One Lambda, Thermo Fisher Scientific) at any time, pre- or post-transplant. T- and B-cell flow crossmatches were negative. Although he was discharged post-transplant with excellent allograft function, he was noted to have worsening respiratory symptoms 4 months post-transplant. He was treated with steroids for presumed rejection. He also had worsening renal function due to tacrolimus-induced thrombotic microangiopathy, which was diagnosed on kidney biopsy. This led to discontinuation of tacrolimus and initiation of cyclosporine at 6 months post-transplant. Kidney biopsy was not performed during the patient’s clinical course; therefore, renal C4d staining and histopathologic assessment for antibody-mediated injury were not available. Consequently, it was not possible to determine whether complement activation extended beyond the lung allograft.

Belatacept, a selective T-cell co-stimulation blocker known to inhibit CD4+ T-cell activation and modulate humoral immune responses, was also added at this time [24].

**Figure 1:**
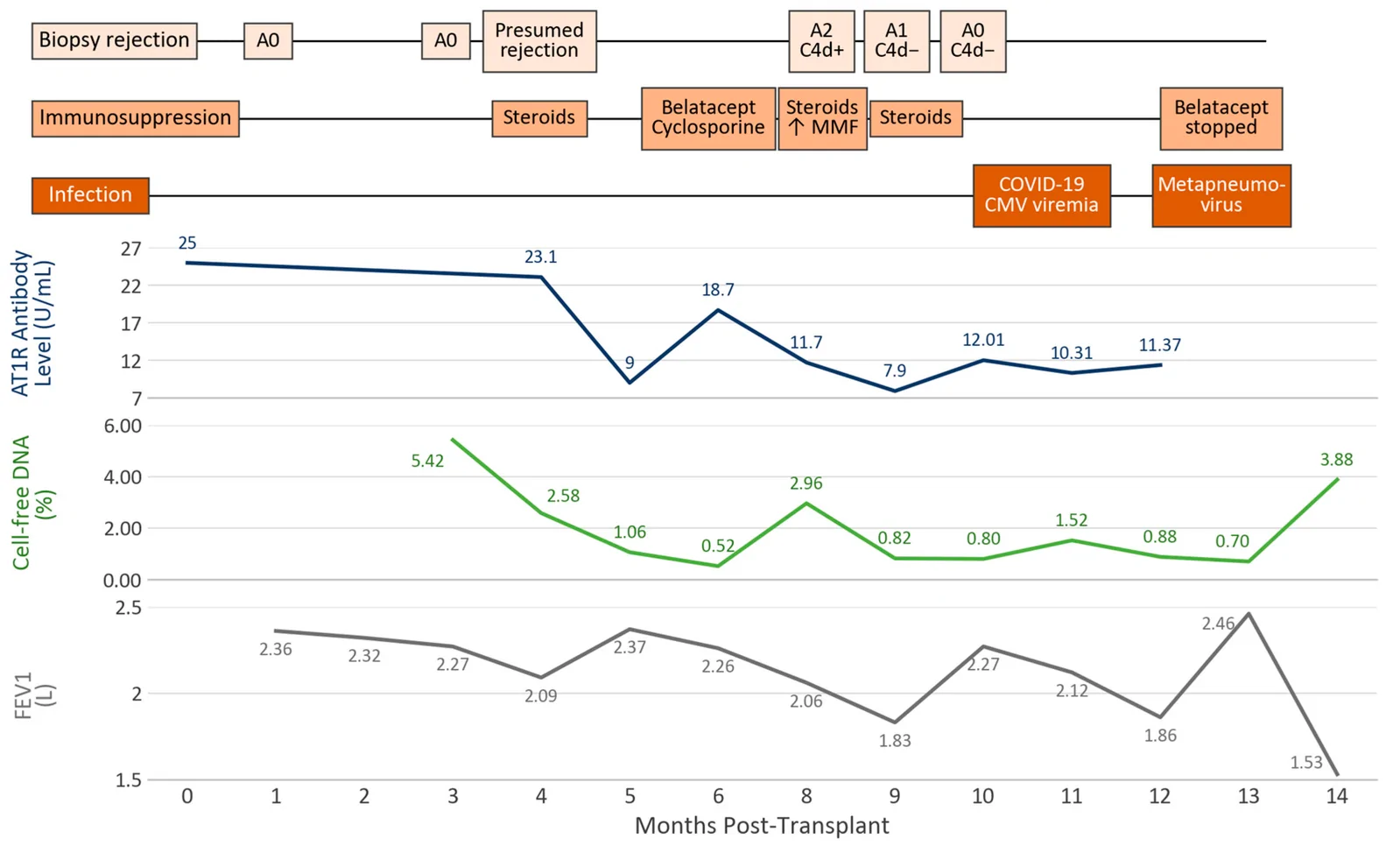
Longitudinal anti-AT1R antibody levels and cell-free DNA levels after lung transplantation, with key clinical events and immunosuppression changes. AT1R antibody levels, cell-free DNA levels, and forced expiratory volume in 1 s (FEV1) are shown for the 14 months after lung transplantation, together with key clinical events and immunosuppression changes. The upward arrow (↑) indicates an increased dose. Abbreviations: AT1R, angiotensin II type 1 receptor; C4d+/C4d−, positive/negative C4d staining; CMV, cytomegalovirus; ISHLT, International Society for Heart and Lung Transplantation; MMF, mycophenolate mofetil. ISHLT lung biopsy grading: A0 = none; A1 = minimal; A2 = mild; A3 = moderate; A4 = severe acute cellular rejection.

**Figure 2:**
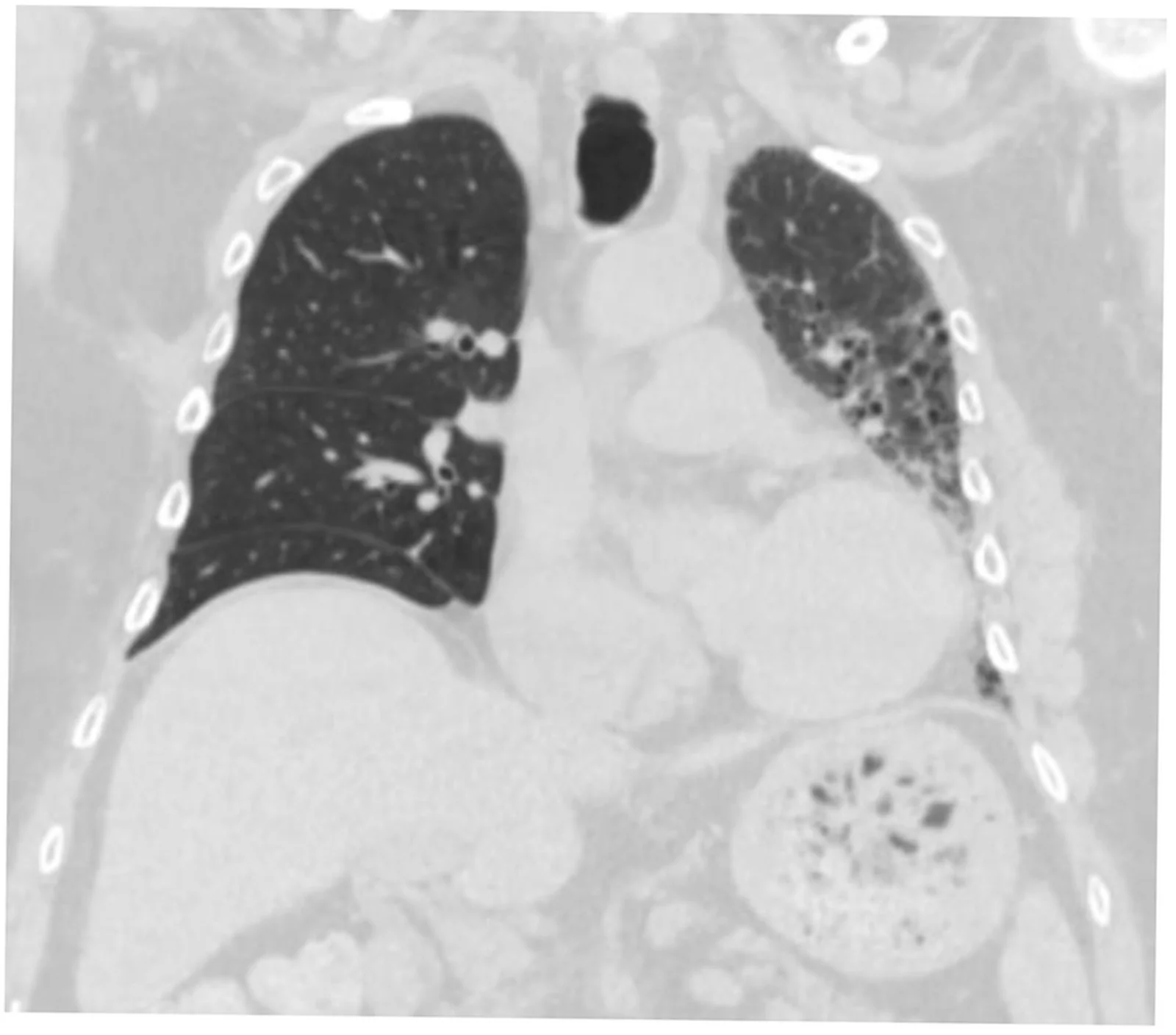
Computed tomography (CT) imaging during the rejection episode shows mild atelectasis of the right lower lobe without consolidation. The transplanted right lung shows mild lower lobe atelectasis, with fibrosis of the native left lung.

**Figure 3:**
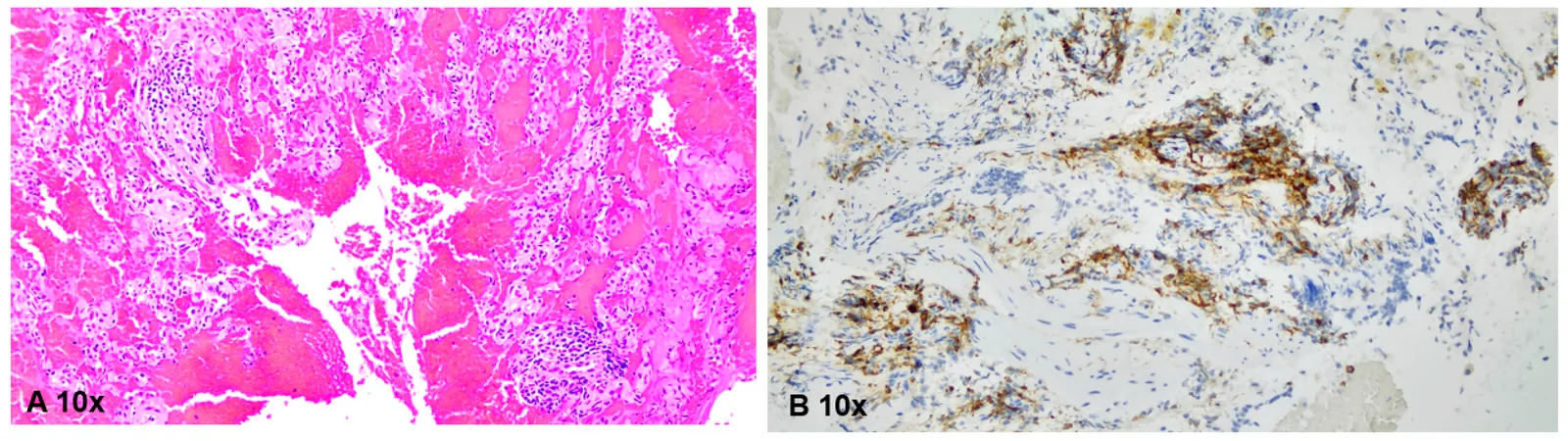
Biopsy evaluations show histological rejection at month 8 post-transplant. (**A**) Hematoxylin and eosin (H & E) staining (10×) of one biopsy fragment showing prominent mononuclear cell infiltrate around multiple vessels and accompanying endothelial inflammation, consistent with acute cellular rejection grade A2. (**B**) C4d immunohistochemistry (10×) showing patchy C4d positivity in the endothelium of alveolar capillaries.

**Figure 4:**
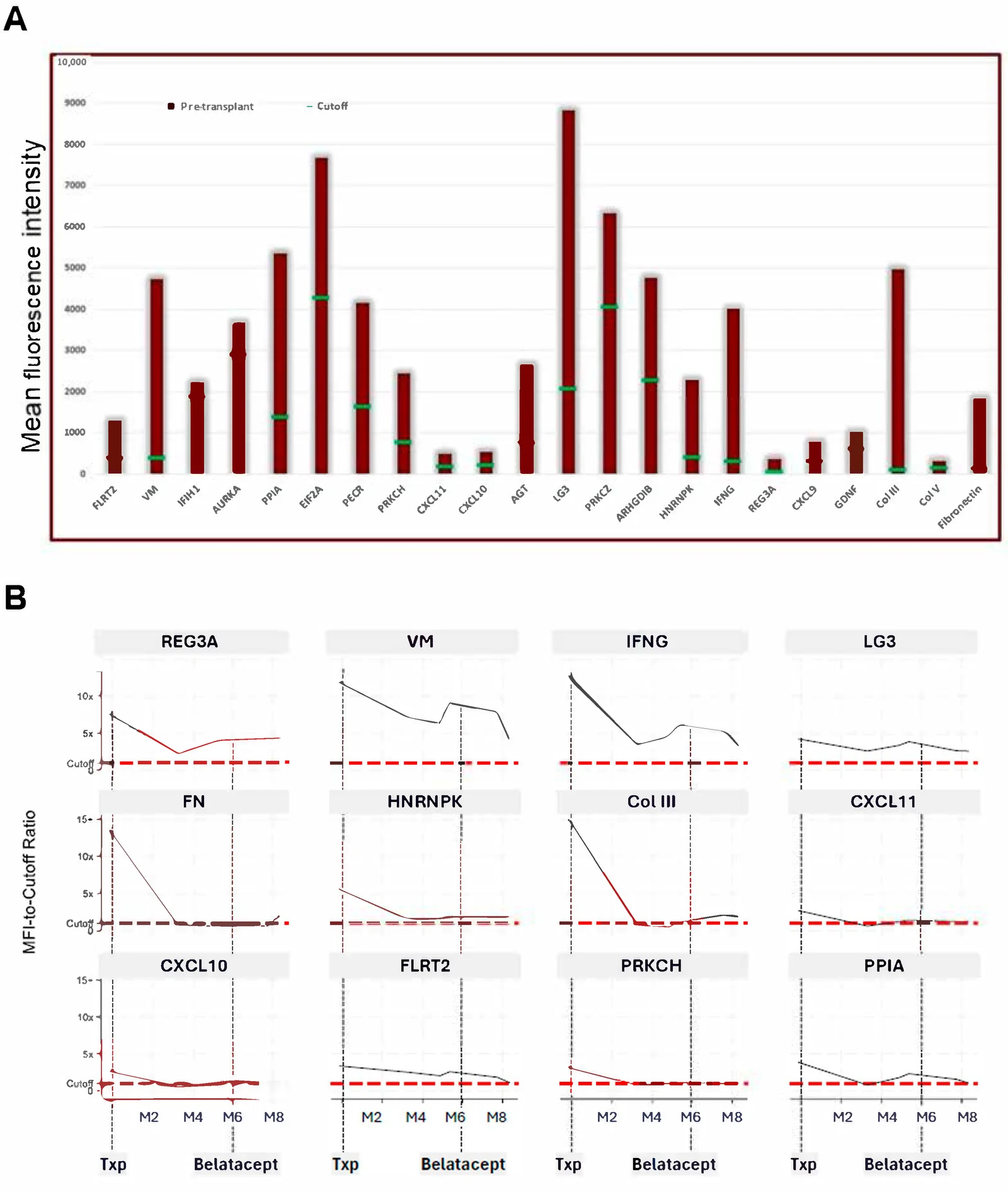
Non-HLA antibody profiles. (**A**) Positive non-HLA antibody profile in the pre-transplant serum of the lung recipient who later developed AMR. The y-axis represents mean fluorescence intensity (MFI) values, which reflect the relative degree of antibody binding detected by the assay. Higher MFI values indicate greater antibody reactivity. Green horizontal bars indicate the cutoff value for each antibody. (**B**) Non-HLA antibody profiles in the pre- and post-transplant sera. After the AMR diagnosis, the levels of the twelve non-HLA antibodies remained above the threshold. The MFI-to-cutoff ratio was calculated for each autoantibody by normalizing the raw MFI against a manufacturer-defined threshold derived from a healthy control cohort. Solid black lines show the MFI-to-cutoff ratio of each antibody over time; the red dashed horizontal line indicates the cutoff (MFI-to-cutoff ratio = 1); and the black dashed vertical lines indicate transplantation (Txp) and the initiation of belatacept. Abbreviations: AGT, angiotensinogen; AMR, antibody-mediated rejection; ARHGDIB, Rho GDP dissociation inhibitor beta; AURKA, aurora kinase A; Col III, collagen III; Col V, collagen V; CXCL9, C-X-C motif chemokine ligand 9; CXCL10, C-X-C motif chemokine ligand 10; CXCL11, C-X-C motif chemokine ligand 11; EIF2A, eukaryotic translation initiation factor 2A; FLRT2, fibronectin leucine rich transmembrane protein 2; FN, fibronectin; GDNF, glial cell line-derived neurotrophic factor; HNRNPK, heterogeneous nuclear ribonucleoprotein K; IFIH1, interferon induced with helicase C domain 1; IFNG, interferon gamma; LG3, C-terminal laminin-like globular domain of perlecan; M, month; PECR, peroxisomal trans-2-enoyl-CoA reductase; PPIA, peptidylprolyl cis-trans isomerase A; PRKCH, protein kinase C eta; PRKCZ, protein kinase C zeta; REG3A, regenerating islet-derived protein 3-alpha; VM, vimentin.

Pulmonary function testing at 8–9 months post-transplant showed declining forced expiratory volume in 1 s (FEV1; 22% change below baseline, see Figure 1) concerning for allograft dysfunction, without signs of infection or consolidation on computed tomography (CT) scan (Figure 2). Combined with bronchoscopy with bronchoalveolar lavage (BAL) and transbronchial biopsy showing endothelial inflammation and positive C4d staining (Figure 3), these findings were concerning for possible clinical AMR by International Society for Heart and Lung Transplantation (ISHLT) criteria [28]. It should be noted that acute cellular rejection (ACR) grade A2 was noted on transbronchial biopsy at this time. In the absence of detectable HLA-DSAs, the rejection episode was attributed to non-HLA antibody-mediated responses, with particular attention to the pre-formed AT1R (25 U/mL) and markedly elevated levels of 23 autoantibodies (>50% of panel reactivity above established cutoffs) (Figure 1 and Figure 4A). The patient was treated with a multi-agent immunosuppressive regimen consisting of escalating prednisone and mycophenolate doses, along with cyclosporine and belatacept (Figure 1). He was also treated with azithromycin due to concern for early CLAD, along with infectious prophylaxis.

Levels of AT1R and other non-HLA autoantibodies were reassessed at multiple intervals during and after this treatment, confirming a sustained reduction in both AT1R antibodies and overall autoantibody levels. Anti-AT1R levels declined by 56% (25 U/mL pre-transplant to 11 U/mL in 1 year).

AT1R antibody testing was not performed at the time of belatacept discontinuation. Therefore, it was not possible to directly assess the antibody level at treatment cessation or evaluate the subsequent trajectory of AT1R antibodies following withdrawal of belatacept.

The patient’s autoantibody specificities decreased from 22 elevated antibodies pre-transplant (59% panel reactivity), to 12 (31%) in post-transplant testing, and eventually to 5 (13%) at 1 year post-transplant. Analysis of MFI-to-cutoff ratios identified a stratification of risk, isolating regenerating islet-derived protein 3-alpha (REG3A), vimentin (VM), interferon gamma (IFNG), and C-terminal fragment of perlecan (LG3) as high-risk targets that exceeded the established thresholds at all timepoints (Figure 4B). REG3A, VM and IFNG remained persistently elevated, with the levels more than four times higher than the threshold (>4.0×) by 8.5 months post-transplant. Cell-free DNA decreased at this time (Figure 1). He noted improved respiratory symptoms and exercise tolerance during office visits, and FEV1 stabilized in the months after this multi-agent rejection treatment; this suggested a clinically significant response (Figure 1). These findings suggest that a multimodal immunosuppression regimen including steroids, mycophenolate, cyclosporine, and belatacept may have effectively contributed to progressive reduction in non-HLA antibody levels. C4d staining was also negative 3 months after the initiation of belatacept, cyclosporine, and increased prednisone and mycophenolate. Intensive monitoring for infectious disease was maintained due to the known infection risks of belatacept and other immunosuppressive medications [25,26,27]. The patient was hospitalized for COVID-19 at month 11 post-transplant. His course was further complicated by increasing cytomegalovirus (CMV) viremia and a subsequent metapneumovirus infection, which ultimately led to the discontinuation of belatacept.

## 3. Discussion

To our knowledge, this is the first reported lung transplant case of C4d-positive histologic antibody-mediated rejection (AMR) associated with multiple non-HLA antibodies, including anti-AT1R, anti-vimentin, and anti-LG3 antibodies, with longitudinal assessment using serial post-transplant serum samples collected over one year. The absence of detectable HLA donor-specific antibodies throughout the clinical course highlights the potential pathogenic role of non-HLA humoral responses in lung allograft injury.

Monitoring for AMR in lung transplant recipients is essential due to the possibility of subclinical AMR, which represents cell-level and tissue-level damage that may accumulate and contribute to chronic lung allograft dysfunction (CLAD). The ISHLT includes clinical allograft dysfunction, histological AMR, C4d staining, donor-specific antibodies, and exclusion of other causes as potentially helpful tests to contribute to diagnosing AMR [28]. In this case, the patient’s respiratory symptoms and spirometry changes were the primary indicators that he had allograft dysfunction, leading to lung biopsy, which illustrated histologic rejection in the absence of HLA-DSAs. Causes including infection were excluded, but because there was coexisting ACR grade A2 on biopsy samples along with these other AMR criteria, this case would be considered “possible clinical AMR” by ISHLT criteria [28].

Growing evidence indicates that non-HLA antibodies can independently contribute to allograft dysfunction despite negative HLA-DSA testing. Antibodies directed against AT1R, endothelin-1 receptor type A (ETAR), vimentin, collagen V, K-alpha-1 tubulin, and LG3 have been associated with acute rejection, chronic lung allograft dysfunction (CLAD), and reduced graft survival across solid-organ transplantation [4,5,6,7,8,9,10,11,12]. In lung transplantation, however, their clinical significance remains incompletely understood, and routine testing is not widely implemented.

Among non-HLA antibodies, anti-AT1R antibodies are among the most extensively studied. Unlike conventional donor-specific antibodies, anti-AT1R antibodies act as agonists, activating the angiotensin II type 1 receptor and triggering intracellular signaling pathways involved in endothelial dysfunction, inflammation, vascular remodeling, and fibrosis [4,11,20]. Several studies have demonstrated that elevated pre-transplant AT1R antibody levels are associated with increased risks of acute rejection, development of de novo HLA-DSA, earlier CLAD, and reduced survival following lung transplantation [4,6,11]. The elevated AT1R antibody levels observed in our patient, together with histologic evidence of AMR, support a potential pathogenic contribution of this antibody to graft injury.

The concurrent presence of anti-vimentin and anti-LG3 antibodies further supports a broader injury-induced autoimmune response. Vimentin is an intracellular cytoskeletal protein that becomes exposed during ischemia–reperfusion injury, apoptosis, and chronic inflammation. Subsequent development of anti-vimentin antibodies has been linked to endothelial activation, complement deposition, chronic rejection, and graft fibrosis [9,21,29,30]. Similarly, LG3, a biologically active fragment of the extracellular matrix proteoglycan perlecan, is released during endothelial injury. Anti-LG3 antibodies have been associated with vascular inflammation, ischemia–reperfusion injury, delayed graft function, and adverse transplant outcomes [12,22,23]. Together, these findings support the concept that tissue injury may expose cryptic self-antigens, break immune tolerance, and promote the development of pathogenic autoantibodies that amplify allograft injury.

Notably, serial measurements demonstrated a reduction in AT1R, anti-vimentin, and anti-LG3 antibody levels following treatment with a multimodal immunosuppressive regimen consisting of belatacept, cyclosporine, mycophenolate mofetil, and increased corticosteroids.

The observed decline in AT1R, anti-vimentin, and anti-LG3 antibody levels following treatment is noteworthy; however, the patient received multiple immunomodulatory agents concurrently, precluding attribution of these changes to any single therapy. Corticosteroids likely contributed through their broad anti-inflammatory and immunosuppressive effects, including inhibition of T-cell activation and cytokine production. Mycophenolate mofetil may have reduced ongoing lymphocyte proliferation and antibody production by blocking purine synthesis in activated T and B cells. Cyclosporine, a calcineurin inhibitor, suppresses T-cell activation and may indirectly attenuate immune responses that support antibody generation. Belatacept inhibits CD28-mediated co-stimulation and has been shown to modulate T-cell-dependent B-cell responses, including suppression of T follicular helper cell differentiation and germinal center formation, thereby potentially affecting alloantibody production [24,25,26,27]. While belatacept has demonstrated benefit in selected cases of antibody-mediated rejection in kidney transplantation, experience in lung transplantation remains limited [24,25,26,27]. Given the simultaneous administration of these therapies, however, the relative contribution of any individual agent to the reduction in non-HLA antibody levels and clinical improvement cannot be determined. Therefore, the observed decline in antibody levels should be interpreted as reflecting the cumulative effects of combination immunosuppressive therapy rather than a specific treatment effect. A key limitation of this report is the inability to distinguish the individual effects of belatacept, cyclosporine, corticosteroids, and mycophenolate mofetil on non-HLA antibody reduction or clinical outcomes.

The patient’s post-transplant course was also notable for recurrent infectious complications, including CMV viremia and COVID-19 infection. These findings are consistent with previous reports describing an increased risk of viral and opportunistic infections in lung transplant recipients receiving belatacept-based immunosuppression [25,26,27]. The observed infectious burden highlights the delicate balance between controlling alloimmune responses and preserving protective antimicrobial immunity.

An additional finding was persistent positivity of REG3A with a slight upward trend during follow-up. REG3A is a well-established biomarker of gastrointestinal graft-versus-host disease and systemic tissue injury [31,32,33]. The persistent elevation of REG3A should be interpreted with caution. Although the patient’s clinical course included episodes of infection and systemic inflammatory activity that may have contributed to elevated REG3A levels [34], the available data do not establish the underlying cause of this finding. REG3A elevation may reflect infection-related immune activation, alloimmune injury, nonspecific inflammatory responses, or a combination of these mechanisms. Therefore, the observed association in this case should be considered hypothesis-generating rather than evidence of a specific pathogenic process.

This case has several important clinical implications. First, it supports growing evidence that AMR can occur in the absence of detectable HLA-DSA and supports consideration of non-HLA antibody testing in selected lung transplant recipients with unexplained graft dysfunction. Second, it highlights the potential contribution of non-HLA antibodies in lung allograft injury occurring in the absence of detectable HLA donor-specific antibodies. However, given the observational nature of a single case report, our findings should be interpreted with caution and should not be viewed as evidence supporting routine non-HLA antibody testing or serial monitoring in all lung transplant recipients. Rather, expanded non-HLA antibody assessment may be considered in selected patients with otherwise unexplained graft dysfunction, particularly when conventional HLA antibody testing is negative and clinical suspicion for antibody-mediated rejection persists. Further studies involving larger patient cohorts are needed to determine the diagnostic and clinical value of non-HLA antibody profiling and longitudinal monitoring in transplant recipients.

Finally, it suggests that belatacept may represent a useful component of combination therapy for non-HLA-mediated AMR, although larger studies are needed to define its efficacy, optimal timing, and safety profile in lung transplantation.

This case is limited in that causal relationships between individual antibodies and graft injury cannot be established from a single case. Furthermore, the patient’s multi-agent immunosuppressive regimen for rejection started due to clinical symptoms before biopsy data could be obtained. He was also found to have coexisting ACR, limiting diagnostic certainty for AMR. In addition, standardized assays, diagnostic thresholds, and consensus guidelines for non-HLA antibody testing remain lacking. Future multicenter studies are needed to define the prevalence, kinetics, and clinical significance of non-HLA antibodies and to establish evidence-based strategies for diagnosis and treatment of non-HLA-mediated AMR in lung transplantation.

## 4. Conclusions

This case demonstrates that C4d-positive antibody-mediated rejection can occur in the absence of detectable HLA donor-specific antibodies and may be associated with non-HLA antibodies including anti-AT1R, anti-vimentin, and anti-LG3 antibodies. Although the temporal association between non-HLA antibody dynamics and graft injury observed in this case is intriguing, it is insufficient to establish causality or clinical utility. Instead, these findings underscore the need for further investigation of non-HLA immune responses in carefully selected patients with suspected donor-specific antibody-negative AMR.

Although the contribution of individual agents cannot be determined, this case also suggests that belatacept may be a useful component of combination immunosuppressive therapy for non-HLA-mediated AMR. Larger studies are needed to clarify the pathogenic significance of non-HLA antibodies and to establish standardized diagnostic and therapeutic approaches in lung transplantation.

## Figures and Tables

**Table 1:** Selected literature review of cases and primary studies concerning non-HLA antibodies in lung transplant recipients.

Author/Study	Year	Organ	Antibody	Findings
Cozzi et al. [4]	2017	Lung	AT1R and ETAR	AMR without pre-transplant HLA-specific Ab
Reinsmoen et al. [11]	2017	Lung	Non-HLA antibodies (AT1R, ETAR) + HLA-specific antibodies	Pre-transplant non-HLA + HLA-specific Ab had poorer post-transplant outcomes
Son et al. [6]	2023	Lung	AT1R	Pre-transplant AT1R Ab associated with early CLAD and shorter survival
Xu et al. [12]	2024	Lung	18 non-HLA antibodies	Association with CLAD
Vinson et al. [13]	2018	Lung	Anti-A1	AMR without HLA-specific Ab
Budding et al. [5]	2015	Lung	AT1R and ETAR	AT1R, ETAR Ab not associated with BOS
Tiriveedhi et al. [14]	2013	Lung	Kα1 tubulin and collagen V	Pre-transplant Ab against self-antigens associated with graft dysfunction
Comins-Boo et al. [15]	2025	Lung	12 non-HLA antibodies	Greater number of non-HLA Ab associated with AMR
Fernandez et al. [16]	2016	Lung	Collagen type I and V, Kα1 tubulin	AMR without pre-transplant HLA-specific Ab
Smith et al. [17]	1995	Lung	Anti-epithelial cell Ab	Pre-transplant anti-epithelial cell Ab associated with decreased graft survival
Goers et al. [18]	2008	Lung	Anti-epithelial cell Ab, Kα1 tubulin	Post-transplant development of anti-epithelial cell Ab and anti-Kα1 tubulin Ab in patients with BOS
Hachem et al. [19]	2012	Lung	Kα1 tubulin and collagen V	Correlation with DSA; patients who cleared Ab after treatment were less likely to develop BOS

Ab, antibodies; AMR, antibody-mediated rejection; AT1R, angiotensin II type 1 receptor; BOS, bronchiolitis obliterans syndrome; CLAD, chronic lung allograft dysfunction; DSA, donor-specific antibodies; ETAR, endothelin-1 receptor type A; HLA, human leukocyte antigen.

## Data Availability

Data are contained within the article.

## References

[1] Young K.A., Ali H.A., Beermann K.J., Reynolds J.M., Snyder L.D. (2021). Lung Transplantation and the Era of the Sensitized Patient. Front. Immunol..

[2] Kobashigawa J., Colvin M., Potena L., Dragun D., Crespo-Leiro M.G., Delgado J.F., Olymbios M., Parameshwar J., Patel J., Reed E. (2018). The management of antibodies in heart transplantation: An ISHLT consensus document. J. Heart Lung Transplant..

[3] Colvin M.M., Cook J.L., Chang P., Francis G., Hsu D.T., Kiernan M.S., Kobashigawa J.A., Lindenfeld J., Masri S.C., Miller D. (2015). Antibody-mediated rejection in cardiac transplantation: Emerging knowledge in diagnosis and management: A scientific statement from the American Heart Association. Circulation.

[4] Cozzi E., Calabrese F., Schiavon M., Feltracco P., Seveso M., Carollo C., Loy M., Cardillo M., Rea F. (2017). Immediate and Catastrophic Antibody-Mediated Rejection in a Lung Transplant Recipient With Anti–Angiotensin II Receptor Type 1 and Anti–Endothelin-1 Receptor Type A Antibodies. Am. J. Transplant..

[5] Budding K., van de Graaf E.A., Hoefnagel T., Kwakkel-van Erp J.M., van Kessel D.A., Dragun D., Hack C.E., Otten H.G. (2015). Anti-ETAR and anti-AT1R autoantibodies are elevated in patients with endstage cystic fibrosis. J. Cyst. Fibros..

[6] Son B.S., Lee H.J., Cho W.H., So M.W., Park J.M., Yeo H.J. (2023). Association of positive pre-transplant angiotensin II type 1 receptor antibodies with clinical outcomes in lung transplant recipients. Transpl. Immunol..

[7] Sureshbabu A., Fleming T., Mohanakumar T. (2020). Autoantibodies in lung transplantation. Transpl. Int..

[8] Yang W., Lecuona E., Wu Q., Liu X., Sun H., Alam H., Nadig S.N., Bharat A. (2023). The role of lung-restricted autoantibodies in the development of primary and chronic graft dysfunction. Front. Transplant..

[9] McQuiston A., Emtiazjoo A., Angel P., Machuca T., Christie J., Atkinson C. (2021). Set Up for Failure: Pre-Existing Autoantibodies in Lung Transplant. Front. Immunol..

[10] Akbarpour M., Wu Q., Liu X., Sun H., Lecuona E., Tomic R., Bhorade S., Mohanakumar T., Bharat A. (2019). Clinical relevance of lung-restricted antibodies in lung transplantation. Hum. Immunol..

[11] Reinsmoen N.L., Mirocha J., Ensor C.R., Marrari M., Chaux G., Levine D.J., Zhang X., Zeevi A. (2017). A 3-Center study reveals new insights into the impact of Non-HLA antibodies on lung transplantation outcome. Transplantation.

[12] Xu Q., Elrefaei M., Taupin J.L., Hitchman K.M.K., Hiho S., Gareau A.J., Iasella C.J., Marrari M., Belousova N., Bettinotti M. (2024). Chronic lung allograft dysfunction is associated with an increased number of non-HLA antibodies. J. Heart Lung Transplant..

[13] Vinson A.D., Rampolla-Selles R., Cooper E.S., Alquist C.R. (2018). Non-Human Leukocyte Antigen Antibody-Mediated Lung Transplant Rejection: The Other Anti-A. Ochsner J..

[14] Tiriveedhi V., Gautam B., Sarma N.J., Askar M., Budev M., Aloush A., Hachem R., Trulock E., Myers B., Patterson A.G. (2013). Pre-transplant antibodies to Kα1 tubulin and collagen-V in lung transplantation: Clinical correlations. J. Heart Lung Transplant..

[15] Comins-Boo A., Mora-Fernández V.M., Padrón-Aunceame P., Toriello-Suárez M., González-López E., Roa-Bautista A., Castro-Hernández C., Iturbe-Fernández D., Cifrián José M., López-Hoyos M. (2025). Non-HLA Antibodies and the Risk of Antibody-mediated Rejection without Donor-specific Anti-HLA Antibodies After Lung Transplantation. Transplant. Proc..

[16] Fernandez R., Chiu S., Raparia K., Garcha P., Farver C., Budev M., Tambur A.R., DeCamp M.M., Budinger S., Perlman H. (2016). Humoral Human Lung Allograft Rejection by Tissue-Restricted Non-HLA Antibodies. Ann. Thorac. Surg..

[17] Smith J.D., Crisp S.J., Dunn M.J., Pomerance A., Yacoub M.H., Rose M.L. (1995). Pre-transplant anti-epithelial cell antibodies and graft failure after single lung transplantation. Transpl. Immunol..

[18] Goers T.A., Ramachandran S., Aloush A., Trulock E., Patterson G.A., Mohanakumar T. (2008). De Novo Production of K-α1 Tubulin-Specific Antibodies: Role in Chronic Lung Allograft Rejection. J. Immunol..

[19] Hachem R.R., Tiriveedhi V., Patterson G.A., Aloush A., Trulock E.P., Mohanakumar T. (2012). Antibodies to K-α 1 Tubulin and Collagen V Are Associated With Chronic Rejection After Lung Transplantation. Am. J. Transplant..

[20] Dragun D., Müller D.N., Bräsen J.H., Fritsche L., Nieminen-Kelhä M., Dechend R., Kintscher U., Rudolph B., Hoebeke J., Eckert D. (2005). Angiotensin II Type 1–Receptor Activating Antibodies in Renal-Allograft Rejection. N. Engl. J. Med..

[21] Rose M.L. (2013). Role of anti-vimentin antibodies in allograft rejection. Hum. Immunol..

[22] Mawad H., Pinard L., Medani S., Chagnon M., Boucquemont J., Turgeon J., Dieudé M., Hamelin K., Rimbaud A.K., Belayachi A. (2023). Hypothermic Perfusion Modifies the Association Between Anti-LG3 Antibodies and Delayed Graft Function in Kidney Recipients. Transpl. Int..

[23] Yang B., Dieudé M., Hamelin K., Hénault-Rondeau M., Patey N., Turgeon J., Lan S., Pomerleau L., Quesnel M., Peng J. (2016). Anti-LG3 Antibodies Aggravate Renal Ischemia–Reperfusion Injury and Long-Term Renal Allograft Dysfunction. Am. J. Transplant..

[24] Kumar D., Raynaud M., Chang J., Reeve J., Yakubu I., Kamal L., Levy M., Bhati C., Kimball P., King A. (2021). Impact of Belatacept Conversion on Renal Function, Histology, and Gene Expression in Kidney Transplant Patients With Chronic Active Antibody-mediated Rejection. Transplantation.

[25] Heldman M.R., Saullo J.L., Menachem B.M., Messina J.A., Arif S., Steinbrink J.M., Tam P.C.K., Carugati M., Wolfe C.R., Baker A.W. (2025). Epidemiology of Infections in Lung Transplant Recipients Treated with Belatacept. Transpl. Infect. Dis..

[26] Vahdatpour C., Saha B., Younis M., Montuoro C., Timofte I., Rackauskas M., Emtiazjoo A. (2024). A case report of a lung transplant recipient receiving belatacept in combination with low dose tacrolimus complicated by progressive multifocal leukoencephalopathy. Respir. Med. Case Rep..

[27] Huang H.J., Schechtman K., Askar M., Bernadt C., Mittler B., Dore P., Witt C., Byers D., Vazquez-Guillamet R., Halverson L. (2022). A pilot randomized controlled trial of de novo belatacept-based immunosuppression following anti-thymocyte globulin induction in lung transplantation. Am. J. Transplant..

[28] Levine D.J., Glanville A.R., Aboyoun C., Belperio J., Benden C., Berry G.J., Hachem R., Hayes D., Neil D., Reinsmoen N.L. (2016). Antibody-mediated rejection of the lung: A consensus report of the International Society for Heart and Lung Transplantation. J. Heart Lung Transplant..

[29] Besarani D., Cerundolo L., Smith J.D., Procter J., Barnardo M.C., Roberts I.S., Friend P.J., Rose M.L., Fuggle S.V. (2014). Role of anti-vimentin antibodies in renal transplantation. Transplantation.

[30] Young R.K., Dale B., Russell S.D., Zachary A.A., Tedford R.J. (2015). Incidence and early outcomes associated with pre-transplant antivimentin antibodies in the cardiac transplantation population. Clin. Transplant..

[31] Ferrara J.L.M., Harris A.C., Greenson J.K., Braun T.M., Holler E., Teshima T., Levine J.E., Choi S.W., Huber E., Landfried K. (2011). Regenerating islet-derived 3-alpha is a biomarker of gastrointestinal graft-versus-host disease. Blood.

[32] Srinagesh H.K., Ferrara J.L.M. (2019). MAGIC biomarkers of acute graft-versus-host disease: Biology and clinical application. Best Pract. Res. Clin. Haematol..

[33] Solán L., Kwon M., Carbonell D., Dorado N., Balsalobre P., Serrano D., Chicano-Lavilla M., Anguita J., Gayoso J., Díez-Martín J.L. (2019). ST2 and REG3α as Predictive Biomarkers After Haploidentical Stem Cell Transplantation Using Post-transplantation High-Dose Cyclophosphamide. Front. Immunol..

[34] Abers M.S., Delmonte O.M., Ricotta E.E., Fintzi J., Fink D.L., de Jesus A.A.A., Zarember K.A., Alehashemi S., Oikonomou V., Desai J.V. (2021). An immune-based biomarker signature is associated with mortality in COVID-19 patients. JCI Insight.

